# Disruption of Microtubule Integrity Initiates Mitosis during CNS Repair

**DOI:** 10.1016/j.devcel.2012.06.002

**Published:** 2012-08-14

**Authors:** Torsten Bossing, Claudia S. Barros, Bettina Fischer, Steven Russell, David Shepherd

**Affiliations:** 1 School of Biological Sciences, Bangor University, Deiniol Road, Bangor LL57 2UW, UK; 2 Cambridge Systems Biology Centre, University of Cambridge, Tennis Court Road, Cambridge CB2 1QR, UK; 3 Department of Genetics, University of Cambridge, Downing Street, Cambridge CB2 3EH, UK

## Abstract

Mechanisms of CNS repair have vital medical implications. We show that traumatic injury to the ventral midline of the embryonic *Drosophila* CNS activates cell divisions to replace lost cells. A pilot screen analyzing transcriptomes of single cells during repair pointed to downregulation of the microtubule-stabilizing GTPase *mitochondrial Rho* (*Miro*) and upregulation of the Jun transcription factor *Jun-related antigen* (*Jra*). Ectopic *Miro* expression can prevent midline divisions after damage, whereas *Miro* depletion destabilizes cortical β-tubulin and increases divisions. Disruption of cortical microtubules, either by chemical depolymerization or by overexpression of monomeric tubulin, triggers ectopic mitosis in the midline and induces *Jra* expression. Conversely, loss of *Jra* renders midline cells unable to replace damaged siblings. Our data indicate that upon injury, the integrity of the microtubule cytoskeleton controls cell division in the CNS midline, triggering extra mitosis to replace lost cells. The conservation of the identified molecules suggests that similar mechanisms may operate in vertebrates.

## Highlights

► CNS midline cells react to injury with ectopic mitosis ► Injury, Miro depletion, and monomeric tubulin expression weaken cortical microtubules ► Microtubule destabilization activates Jun expression ► Jun expression is essential to release damaged midline cells from G2 arrest

## Teaser

Bossing et al. show that injury to embryonic *Drosophila* CNS releases ventral midline cells from G2 arrest, resulting in extra mitoses to replace lost cells. Depolymerisation of cortical microtubules, enhanced by decreased expression of Miro (Mitochondrial Rho) triggers expression of the transcriptional factor Jun, which is essential for damage-induced mitosis.

## Introduction

The mechanisms regulating mitotic activation of neural stem cells during brain repair or disease are of key interest (Liu et al., 2009). Focal ischemias induce the proliferation of neural stem cells in the subventricular zone and stimulate forebrain neurogenesis. Similarly, traumatic brain injuries trigger adult neural stem cell divisions in the dentate gyrus (Kernie and Parent, 2010). Therefore, damage-induced proliferation of neural stem cells after stroke or mechanical injury may enhance posttraumatic recovery (Liu et al., 2009). Increased cell divisions, however, may also be harmful. For example, following temporal lobe epileptic seizures, neural stem cell divisions in the dentate gyrus show a robust increase, which may exacerbate subsequent seizures (Scharfman and Gray, 2007). Brains from patients suffering from tauopathies also exhibit ectopic cell cycle activation in differentiated neurons, which exacerbates neuronal death (Busser et al., 1998; Khurana and Feany, 2007). It is increasingly evident that damage-induced cell cycle activation, either beneficial or harmful, plays an important role in CNS repair and the pathology of neurologic diseases. Despite their importance, the mechanisms triggering damage-induced mitosis are unknown.

To gain insight into the mechanisms of damage-induced divisions, we took advantage of the ventral midline in the embryonic CNS of *Drosophila*. The ventral midline is the functional analog of the vertebrate floorplate and serves as a model system for axonal guidance (Arendt and Nübler-Jung, 1999). In *Drosophila*, every trunk segment has six to ten midline precursors that generate approximately 20 neurons and glial cells (Bossing and Technau, 1994). Unlike neural precursors (neuroblasts) that delaminate as single cells, midline cells segregate from the ectoderm as a continuous strip of cells and most cells go through only one, nearly simultaneous, division during early development (Bossing and Technau, 1994; Dong and Jacobs, 1997). The small number of divisions, the limited number of cells and a complete knowledge of their lineage makes the ventral midline an attractive tissue to study damage-induced divisions.

We show that damage releases early ventral midline cells from G2 arrest inducing extra divisions that replace damaged cells. An exploratory microarray analysis of the transcriptome of single cells removed from living embryos shows that upon damage, *mitochondrial Rho* (*Miro*) is the most downregulated transcript and *Jun-related antigen* (*Jra*) is one of the highest upregulated transcripts. Our functional analysis reveals that midline targeted expression of Miro can prevent damage-induced divisions, while uniform depletion of Miro induces ectopic divisions only at the ventral midline. Moreover, uniform depletion of Miro, mechanical damage at the midline, ectopic midline expression of monomeric α-tubulin and injection of microtubule depolymerising drugs decreases dramatically the amount of β-tubulin at the cell cortex and results in extra midline divisions. Midline expression of α-tubulin initiates the expression of *Jra*, which is essential for midline cells to enter into damage-induced mitosis. Our data present a mechanism whereby injury induces cell divisions at the damage site to replace lost cells.

## Results and Discussion

### Damage at the Ventral Midline Releases Midline Cells from G2 Arrest

**Figure 1:**
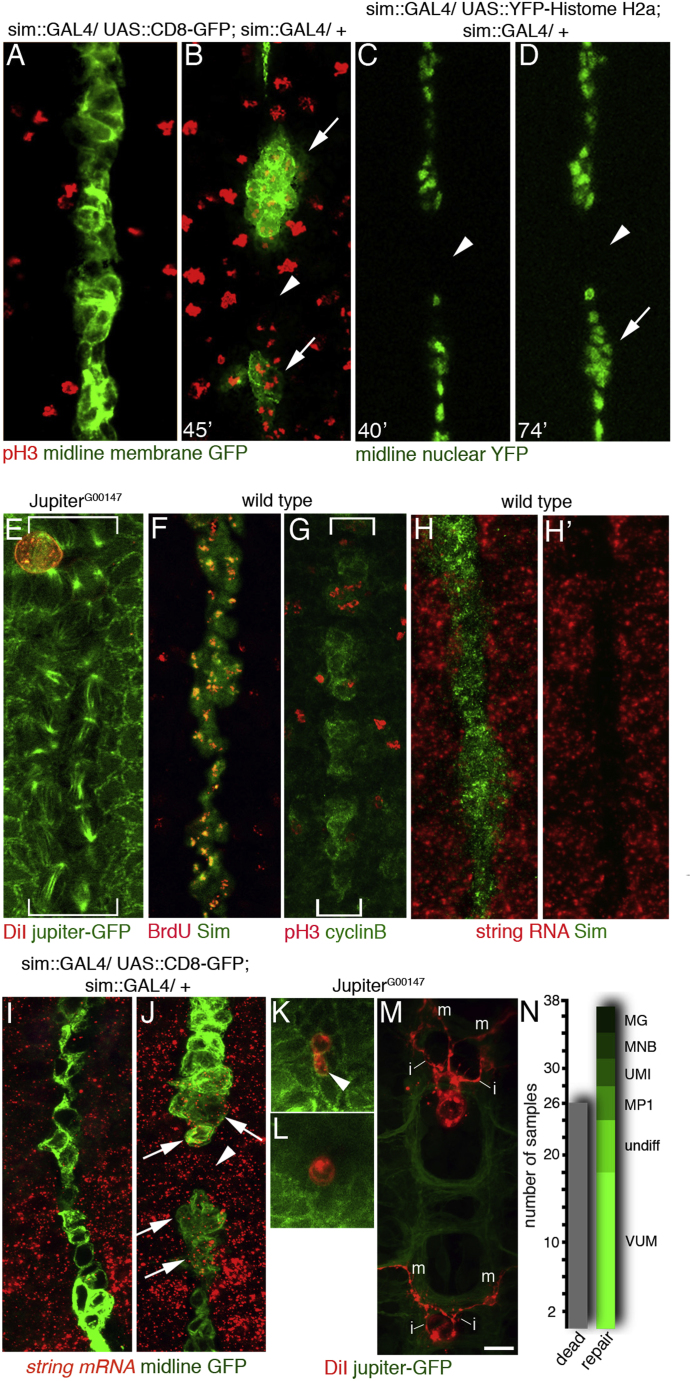
Damage at the Ventral Midline in *Drosophila* Embryos Releases Midline Cells from G2 Arrest Genotypes listed at top of panels. Brackets indicate midline. Ventral views, anterior up. Scale, 6 μm. (A–D) Midline cells (green) in early stage 11 embryos do not divide (A). After mass removal of cells (arrowheads, B–D), adjacent midline cells enter into mitosis (arrows, B, D). Dividing cells are labeled with phosphoHistone H3 antibodies (red, pH3). Time in minutes after ablation. (C) and (D) show different time points of the same embryo. (E) One DiI-labeled midline precursor (red) in *Jupiter*^*G00147*^ embryos (green) in which microtubules are outlined by GFP. All midline cells divide nearly simultaneously shortly after gastrulation. (F) After completion of the initial division, all midline cells (green, Single minded antibody) enter into S phase (red, BrdU). (G) At early stage 13 cyclinB (green) strongly accumulates in midline cells and a small subset of cells divide again (red). (H and H’) For 3.5 hr after the first division, midline cells (green) are devoid of *string* mRNA (red), which encodes for cdc25 phosphatase required for entry into mitosis. H’ shows only *string* mRNA. (I) Midline cells in early stage 11 do not express *string* mRNA (red). (J) *string* is transcribed in midline cells (arrows) after mechanical damage (arrowhead). (K–M) Time sequence of midline repair. Two siblings are generated by the initial division of one labeled midline precursor (red, DiI, K). Ablation of one sibling (arrowhead, K), results in division of the surviving sibling (L) and replacement of the lost sibling (bottom clone, M). A second labeled precursor in the same embryo was left undisturbed (top clone, M). Both precursors gave rise to a VUM clone consisting of one interneuron and one motorneurons. i, interneuronal projection; m, motorneuronal axon. (N) Ablations of midline siblings cause replacement of ablated sibling (green bar) or cell death (gray bar). Replacement always results in clonal types previously described in wild-type. Clonal types are: VUM, ventral unpaired median neurons; MP1, midline precursor 1 neurons; UMI, unpaired median interneurons; MNB, median neuroblast; MG, midline glia; undiff, two round cells with no differentiation. See also Figure S1 and Movies S1, S2, and S3.

To gain insight into the signaling mechanisms involved in damage-induced division, we mechanically damaged the *Drosophila* embryonic CNS at mid stage 10 by removing between 4 and 15 cells (mass ablation) with a microcapillary. We primarily removed midline cells but often neuroblasts were also damaged or removed. In 85% (6/7) of embryos, cell removal from the center of the CNS results in the phosphorylation of Histone H3 Serine 10 (phospho S10; pH3) in cells at the wound, indicating entry into division (Figures 1A and 1B). Cell loss results in collapse of the lateral CNS, which is formed by neuroblasts, toward the midline. Neuroblasts are highly mitotically active (Egger et al., 2008), making it difficult to discern extra divisions induced by trauma. Yet, additional divisions are readily detected in the center of the CNS at the ventral midline. The midline is mitotically quiescent during most of embryogenesis (Figure 1A) (Jacobs, 2000). Mass ablations of midline cells expressing the nuclear marker Histone H2a-YFP confirm additional cell divisions at the damage site (6/9 embryos; Figures 1C and 1D). Between one and four midline cells bordering the injury divided 35–90 min after damage. We never observed dividing midline cells outside the wound area. The number of mitotic cells in H2a-YFP expressing embryos is less than that observed by phosphorylation of Histone H3, which may indicate that not all cells entering into M-phase complete mitosis.

Cells with the ability to replace damaged tissue, such as regenerative cells in Hydra and Newt, regenerating hepatocytes and hippocampal neural stem cells, preferentially arrest in the G2 phase of the cell cycle (Dübel and Schaller, 1990; Galvin et al., 2008; Holstein et al., 1991; Michalopoulos and DeFrances, 1997; Schmidt and David, 1986; Tanaka et al., 1997). We analyzed the divisions of midline cells in situ (Figures 1E–1H’) and in vivo (Movies S1 and S2 available online). All midline cells divide shortly after gastrulation (Figure 1E) and enter into mitotic quiescence for the next 3.5 hr, until mid stage 11. Immediately after the initial division, midline cells go through another S phase and begin accumulating cyclin B (Figures 1F and 1G). The lack of *string* mRNA, the cdc25 ortholog essential for entry into mitosis (Lehner, 1991), shows that early midline cells are arrested in G2 (Figures 1H and 1H’). Damage at the midline induces expression of *string* in cells at the wound, releasing the cells from G2 arrest (5/9 embryos; Figures 1I and 1J). In conclusion, cells of the ventral midline are released from G2 arrest by traumatic injury.

### Undifferentiated Midline Cells Can Replace Sibling Cells Lost by Damage

The developmental lineage of midline precursors is well described (Bossing and Technau, 1994), allowing us to determine if midline cells born due to damage differentiate into neurons or glia. During germband elongation (stage 10) we used a microcapillary to ablate one of two siblings derived from the division of a single DiI labeled midline precursor cell (Figures 1K and 1L). In 41% (n = 63) of ablations the undamaged sibling cell does not divide and dies after germband shortening. However, in 59% of ablations the majority of undamaged siblings divide again and differentiate into a neuron or glial cell (Figures 1M and 1N). The type of progeny depends on the original lineage affected. We do not detect any lineage crossovers, i.e., ablations result in clone types previously described in wild-type embryos (Bossing and Technau, 1994). A minority of survivors divide but do not show any morphologic differentiation (n = 9.5%). The time between ablation and division of the surviving cell varies between 34 and 187 min (mean, 91 min). The fluctuation in timing and the high frequency of cell death may result from varying degrees of damage induced by the ablation.

### Differentiated Midline Cells Cannot Replace Damaged Siblings

We tested whether injured midline cells can re-enter mitosis throughout development by ablating cells after axon outgrowth in different subsets of midline cells (n = 10). In contrast to undifferentiated midline cells, none of the surviving differentiated cells divided again (Figures S1A–S1C). Similarly, ablation of neuroblast precursors in the lateral CNS of early embryos does not result in mitotic activation of the surviving sibling, the ganglion mother cell, to replace the neuroblast (Choksi et al., 2006). Our results show that undifferentiated midline siblings, but not differentiated midline cells, react to injury with additional mitosis. Interestingly, undifferentiated midline cells share a membrane connection, which is lost at the onset of axonal outgrowth (Figures S1D–S1K). Mechanical breakage of this connection results in the death of both siblings (n = 8, data not shown). The function of this connection in mitotic induction after damage is unclear.

Our data point to signaling between the damaged and surviving cells. Midline cells express a battery of signaling molecules and receptors with Hedgehog, Wingless, Notch, and EGF (Spitz) signaling controlling midline cell determination but not the number of divisions (Bossing and Brand, 2006; Stemerdink and Jacobs, 1997; Wheeler et al., 2008). One possibility is that midline cells are prevented from entry into mitosis by contact inhibition. This is unlikely because in most cases, approximately 10% of the membrane of the ablated cell remained in contact with the sibling cell, and ablations do not create persistent gaps between midline cells and ectoderm (Figures 1K and 1L). Nevertheless, we tested signaling via integrin and cadherin, cell contact molecules expressed in the midline during early embryogenesis. We examined midline cell division in embryos maternally and zygotically mutant for the β-integrin subunit, *myospheroid*. The loss of functional integrin heterodimers does not affect the division pattern in the ventral midline (data not shown). We compromised cadherin-based cell adhesion by injection of EGTA (Figures S2A, S2B, S2E, and S2F) or by ectopic expression of Crumbs (Klebes and Knust, 2000) in midline cells (*rho-GAL4/UAS-Crumbs*, data not shown). Interfering with cadherin adhesion results in the loss of midline contact with the ectoderm, but no extra divisions were detected.

### Depletion of *Mitochondrial Rho* Activates Mitosis in Midline Cells

Since testing known cell signaling pathways did not reveal candidate molecules involved in the activation of midline cell division, we chose to compare the transcriptomes of DiI labeled single ablated midline cells with the single surviving siblings that react to damage with an extra division (see Experimental Procedures).

**Figure 2:**
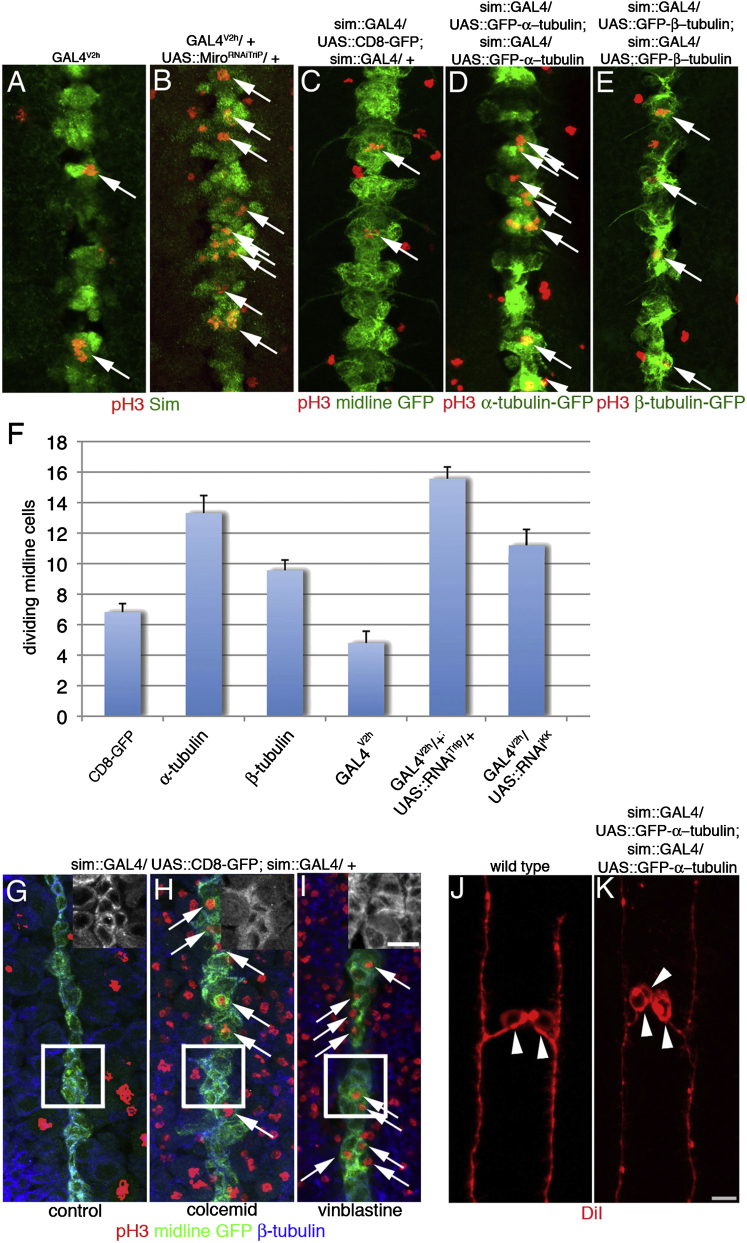
Disruption of Microtubules Triggers Cell Divisions Genotypes listed at top of panel. Ventral views, anterior up. Bar, 6 μm. (A) In stage 13 embryos, a minority of midline cells (green) divide (arrows, red, pH3). (B) Depletion of *Miro* by expression of UAS::MiroRNAi in all tissues results in increased cell divisions in midline cells only. (C–E) Expression of GFP (green, C) does not increase mitosis (arrows, red, pH3) in stage 13 embryos but midline expression of α-tubulin (green, D) and, to a lesser degree, β-tubulin (green, E), triggers extra mitosis. (F) Number of dividing midline cells in all trunk segments of early stage 13 embryos. ANOVA, bars represent SEM. CD8::GFP, *sim::GAL4/ UAS::CD8-GFP* (n = 39)*/ sim::GAL4/ +*. α-tubulin, *UAS::GFP-α−tubulin/ sim::GAL4; UAS::GFP-α-tubulin/ sim::GAL4* (n = 35). β-tubulin, *UAS::GFP-β−tubulin/ sim::GAL4; UAS::GFP-β-tubulin/ sim::GAL4* (n = 36). *GAL4*^*V2h*^ (n = 10). *Gal4*^*V2h*^*/+; UAS::MiroRNA*^*TRIP02775*^*/+* (n = 16). *Gal4*^*V2h*^*/UAS::MiroRNA*^*KK106683*^ (n = 23). Error bars: SEM. (G) In water-injected stage 10 embryos, midline cells (green) do not divide (red) and show a cortical accumulation of β-tubulin (blue). (H and I) Partial depolymerization of microtubules in stage 10 embryos injected with colcemid (n = 14) or vinblastine (n = 27) drives midline cells into division. Insets show higher magnification of boxed area and β-tubulin only. (I) Midline precursor 1 (MP1) in wild-type gives rise to two MP1-neurons (arrowheads). (J) Midline expression of α-tubulin results in extra MP1-neurons (arrowheads) generated by one precursor. See also Figures S2 and S3.

**Figure 3:**
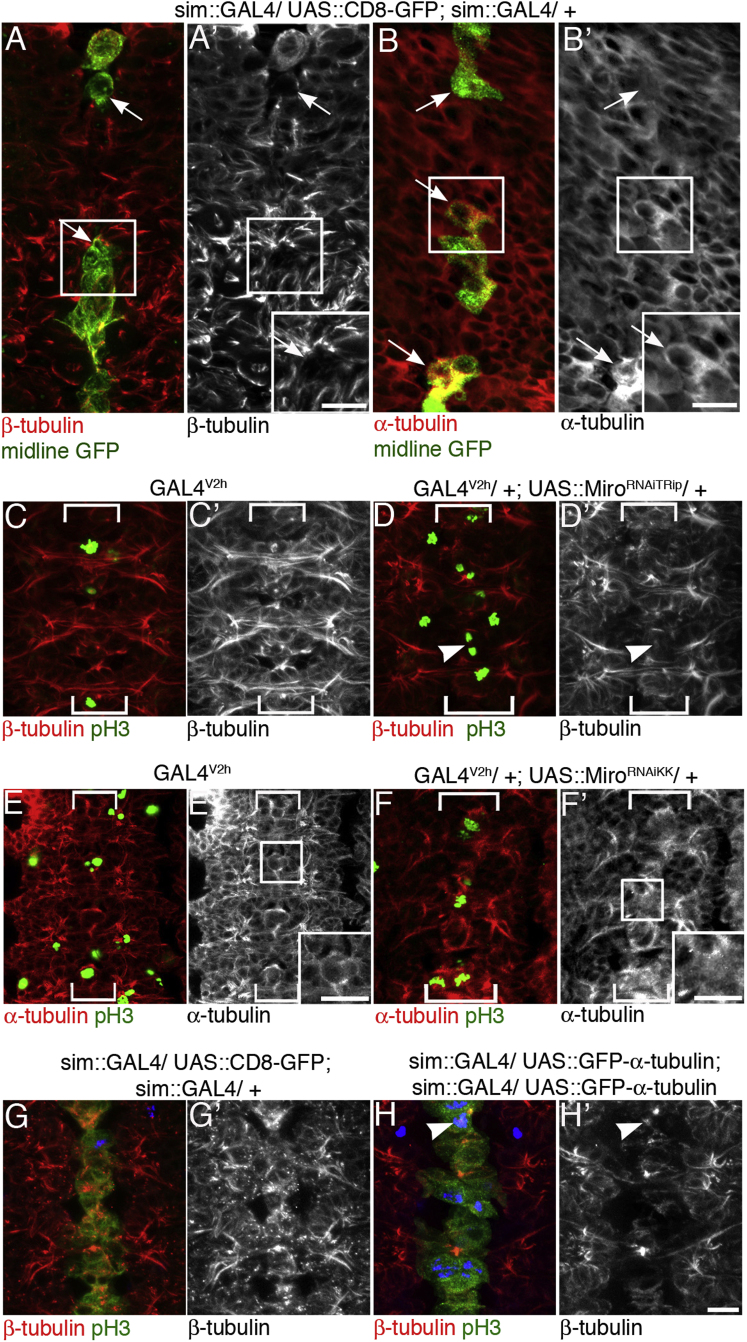
Cortical β-Tubulin Is Reduced by Mechanical Damage, Miro Depletion, and Expression of α-Tubulin Genotypes are listed on top of panels. Insets show higher magnifications of boxed area. A’–H’ shows tubulin only. Ventral views, anterior up. Bar, 6 μm. (A and A’) After mechanical damage, midline cells (green) at the wound (arrow) show a near loss of β-tubulin (red, arrows). (B and B’) Mechanical damage at the ventral midline (green) does not result in decreased α-tubulin staining (red) in cells at the damage site (arrows). Note, the antibody against β-tubulin recognizes mainly cortical tubulin whereas anti-α-tubulin outlines tubulin in cytoplasm and cortex. (C and C’) Midline cells show a robust staining against β-tubulin. (D and D’) Depletion of Miro by RNAi expression throughout the embryo results in a decrease in β-tubulin and an increased mitosis limited to the ventral midline. Depletion of Miro affects cortical tubulin but leaves the mitotic spindle and midbody (arrowhead) intact. (E–F’) In contrast to β-tubulin, the ubiquitous embryonic depletion of Miro (F, F’) leads to an increase of cytoplasmic α-tubulin (green) when compared to controls (E, E’). (G and G’) GFP expressing midline cells (green) show a robust β-tubulin stain. (H and H’) Expression of α-tubulin in midline cells reduces cortical β-tubulin but does not affect the spindle (arrowhead). See also Figure S4.

In our pilot study, *Miro* was the most downregulated transcript (T.B. et al., unpublished data). *Miro*, encodes a widely conserved atypical GTPase, regulating mitochondrial transport and microtubule stabilization (Guo et al., 2005). If downregulation of Miro permits midline cells to enter into divisions, we would expect more progeny per midline precursor in *Miro* mutants. Single labeled midline precursors in *Miro*^*SD32*^ mutants do not, however, divide more often than wild-type precursors (data not shown). This is likely due to a maternal contribution of *Miro* RNA (www.flyexpress.net) and we therefore lowered the amount of *Miro* RNA by ubiquitously expressing *UAS::Miro*^*RNAiTRIPiJF02775*^ or *UAS::Miro*^*RNAiKKi106683*^ with a maternal GAL4 driver (*GAL4*^*V2h*^). Strikingly, embryos from mothers expressing either of the *UAS::Miro*^*RNAi*^ constructs show an increase in midline cell division at the end of germband shortening (Figures 2A, 2B, 2F, and 3C–3F). Compared to control embryos (*GAL4*^*V2h*^) with an average of 4.8 mitotic cells in all 10 trunk neuromeres, the number of midline cell divisions in embryos depleted of Miro increases to an average of 15.6 cells (*UAS::Miro*^*RNAiTRIPJF02775*^) or 11.1 cells (*UAS::Miro*^*RNAiKK106683*^; Figure 2F). If *Miro* downregulation is essential for damage-induced division, midline targeted upregulation may block these divisions. Indeed, in contrast to wild-type embryos, ablations in early stage 11 embryos with midline Miro expression can result in undamaged cells which survive but neither divide nor differentiate (16%, n = 13; Figure S4). Yet, most ablations result in repair (46%) or death (38%). The low percentage of embryos with no repair may indicate that the expression of Miro in most surviving siblings is not high enough to prevent repair.

### Disruption of the Microtubule Cytoskeleton Triggers Ectopic Mitosis in Midline Cells

Miro affects microtubule bundling and transport of mitochondria along microtubules (Guo et al., 2005). The size of midline cells (5 μm) makes it unlikely that mitochondrial transport is a crucial factor for their development. To test if disrupting the microtubule cytoskeleton can initiate ectopic divisions, we expressed tubulin monomers in midline cells. In stage 12/13 embryos expressing midline GFP we find an average of 6.8 dividing midline cells in all 10 trunk neuromeres (Figures 2C and 2F). However, midline expression of α-tubulin nearly doubles the number of proliferating cells to an average of 13.3 cells (Figures 2D and 2F). Expression of β-tubulin only slightly increases the number of mitotic midline cells to 9.6 (Figures 2E and 2F). While targeted GFP and β-tubulin are expressed from stage 11, we only detect α-tubulin midline expression from stage 12. This delayed expression precludes an analysis of the role of microtubule integrity at earlier stages. To overcome this problem we injected the microtubule depolymerizing drugs Colcemid and Vinblastine. Injection of either drug results in microtubule depolymerization in early embryos (stage 10/11) and allows midline cells to enter mitosis during germband elongation (Figures 2G–2I). Compared to water injected embryos with an average of six mitotically active cells, 50 min after injection of either Colcemid (n = 14) or Vinblastine (n = 27) an average of 14 (± 1.2) or 12 (± 0.8) midline cells stain positive for the mitotic marker pH3. Midline cells entering into an extra mitosis are limited to five segments around the site of injection indicating that both drugs show limited diffusion. Colcemid and Vinblastine injections also cause mitotic arrest in nonmidline cells (Figures 2G–2I) and the loss of midline cell ectodermal connections (Figures S2C, S2D, S2G). Unlike microtubule depolymerization, actin depolymerization by Latrunculin A injection does not increase the number of mitotic midline cells (Figures S3A, S3B, S3E, and S3F). In addition, microtubule stabilization via Taxol injection (Figures S3D and S3H) fails to release midline cells from G2 arrest.

To confirm our results, we labeled single midline precursors expressing α-tubulin and examined their progeny. At stage 16/17 in wild-type embryos, most midline clones consist of two cells (75%), some clones have more than four cells (24.5%) but three-cell clones are rare (0.5%) (n = 540) (Bossing and Technau, 1994). Yet, midline precursors expressing α-tubulin often give rise to three daughter cells (38%, n = 29) indicating that one of the two midline siblings divides again. The extra sibling differentiates in accordance with its lineage (Figures 2J and 2K). Some midline precursors still generate only two cells (44%) and a few precursors produce more than four cells (18%). We conclude that midline targeted expression of α-tubulin is able to drive midline cells into division. The delayed onset of α-tubulin expression, shortly before midline cells lose their repair capacity, most likely limits the number of extra divisions. Our data suggest that changes in the microtubule cytoskeleton generated by tubulin expression or by chemical depolymerization allow midline cells to enter into extra mitosis.

### Depletion of β-Tubulin Triggers Extra Mitosis in Midline Cells

Since mechanical damage, Miro depletion, microtubules depolymerizing drugs and expression of α-tubulin all trigger midline cell divisions, we tested if all these treatments induce a similar change in the microtubule cytoskeleton. We stained embryos damaged at the midline for β-tubulin and α-tubulin. In embryos fixed 10–30 min after midline damage we find a near complete loss of β-tubulin in cells at the damage site (7/12 embryos, Figures 3A and 3A’). In contrast, α-tubulin is occasionally increased (3/12 embryos) but most often shows no changes after damage (Figures 3B and 3B’). We also noted that the antibody against β-tubulin predominantly labels the cell cortex whereas α-tubulin is mainly cytoplasmic (Figures 3A’ and 3B’). Ubiquitous depletion of Miro in the embryo also results in decreased β-tubulin (Figures 3C and 3D’). As with the extra mitosis, this decrease in β-tubulin is mainly limited to cells at the midline. We also observe an increase in cytoplasmic α-tubulin in Miro depleted embryos (Figures 3E and 3F’). Midline expression of α-tubulin nearly abolishes β-tubulin accumulation in midline cells (Figures 3G and 3H’) and injection of colcemid or vinblastine (Figures 2H and 3I) reduces cortical β-tubulin accumulation. We conclude that mechanical damage results in a decrease in cortical β-tubulin and these changes can be mimicked by Miro depletion, α-tubulin expression, and microtubule depolymerizing drugs.

### *Jun-Related Antigen* Is Essential for Damage-Induced Cell Divisions

**Figure 4:**
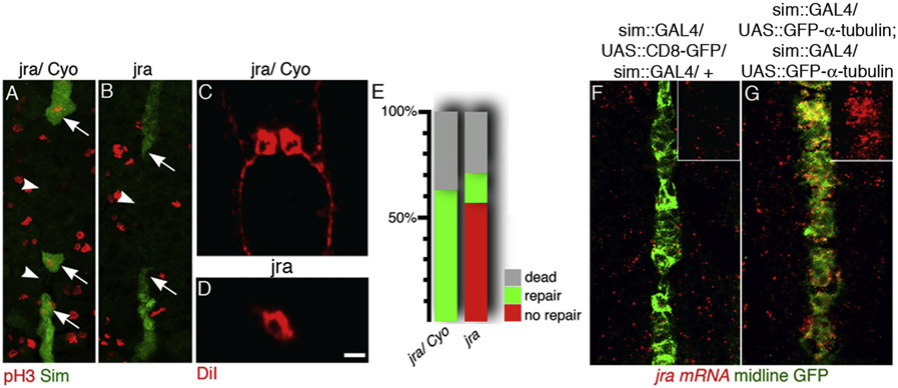
*Jra*, the *Drosophila Jun* Ortholog, Is Essential for Midline Repair Genotypes are listed on top of panels. Ventral views, anterior up. Bar 5 μm. (A and B) In heterozygous embryos (A), but not in *Jra* mutant embryos (B), removal of midline cells (green, arrowhead marks gap) triggers mitosis (arrows; pH3, red). (C) In heterozygous embryos, ablation of midline siblings leads to replacement of the ablated cell. (D) After ablation of one sibling in *Jra* mutants, the surviving sibling does not divide or differentiate. (E) Replacement of ablated sibling cells in heterozygous (*Jra*^*IA109*^*/Cyo*, n = 21) and mutant (*Jra*^*IA109*^*/ Jra*^*IA109*^, n = 14) embryos. No repair, undamaged sibling does not divide; repair, undamaged sibling divides; dead, undamaged sibling dies. (F and G) At stage 13, in wild-type, *jra* mRNA (red) is not detectable in midline cells (green, F) but midline expression of α-tubulin activates *jra* transcription (red, G). Insets only show the red channel at the midline. See also Figure S4.

*Jun* is a transcription factor found to be upregulated in vertebrate brain injury (Raivich and Behrens, 2006). Interestingly we observed upregulation of the *Drosophila Jun* ortholog, *Jra*, in our exploratory microarray analysis (T.B. et al., unpublished data). To test if *Jra* is necessary for damage-induced division, we first performed mass ablation of midline cells in *Jra*^*IA109*^ mutants. In heterozygous embryos, tissue repair is still observed (4/9; Figure 4A) but in *Jra* homozygous mutants injury no longer activates midline cell division (0/5; Figure 4B). Without damage, we detect no significant changes in midline cell divisions between heterozygous and homozygous *Jra* mutant embryos (Figures S4D–S4F). We next ablated single cells in heterozygous and homozygous *Jra* mutant embryos. In heterozygous embryos, single ablated sibling cells were mostly replaced (62%, n = 21; Figures 4C and 4E); however, in *Jra* homozygotes the replacement of ablated single cells is the exception (14%). The majority of surviving siblings do not divide or differentiate (57%, n = 14; Figures 4D and 4E). Since midline expression of α-tubulin drives midline cells into division, we tested if α-tubulin expression is sufficient to switch on *Jra* transcription. Indeed, midline targeted α-tubulin induces ectopic *Jra* expression (Figures 4F and 4G).

The upregulation of *Jun* in vertebrate brains is often linked to apoptosis, but during axonal injury in motorneurons *Jun* is also needed to accelerate axonal regeneration (Raivich et al., 2004). We show that during midline repair, *Jra* does not promote apoptosis since after ablation we see no increase in cell death of survivors in *Jra* mutants (29%) as compared to wild-type embryos (41%). However, *Jra* is necessary for entry of surviving sibling cells into mitosis after damage. Our results suggest that upregulation of *Jra* may be a consequence of the microtubule disruption caused by damage.

### Conclusions

We demonstrate that traumatic injury to undifferentiated ventral midline cells in *Drosophila* triggers extra mitosis at the midline immediately adjacent to the wound. The newly dividing cells replace lost cells. Early midline cells are arrested in the G2 phase of the cell cycle, an arrest which may be crucial for the repair capacity of the midline. In mice, switching cell cycle arrest from G1 to G2 can change a nonregenerating animal into a regenerating one (Bedelbaeva et al., 2010) and many cells with regenerative capacity are arrested in G2 (Dübel and Schaller, 1990; Galvin et al., 2008; Holstein et al., 1991; Michalopoulos and DeFrances, 1997; Schmidt and David, 1986; Tanaka et al., 1997).

The pathway we describe was uncovered by damaging embryos and might be used as a repair mechanism in various other tissues in different animals. However, we believe that during *Drosophila* embryogenesis it may serve as a cell counting mechanism, maintaining tissue homeostasis because only the cells adjacent to the wound undergo an additional mitosis, often resulting in an incomplete repair. During embryogenesis, the number of midline precursors per segment varies between six and ten, with seven precursors generating a full complement of midline cells. Surplus cells are eliminated by apoptosis (Bergmann et al., 2002; Bossing and Technau, 1994). It is tempting to speculate that in segments with only six precursors, signaling between midline siblings may activate divisions to generate additional cells. A mechanism to control cell number in the ventral midline may have evolved to safeguard the proper organization of the developing CNS as midline signaling directs cell fate determination and axonal guidance (Arendt and Nübler-Jung, 1999).

Mechanical damage can deplete β-tubulin in cells at the damaged site, and we can mimic this disruption by *Miro* depletion, α-tubulin expression and microtubule depolymerizing drugs. In most cases, these disruptions do not change α-tubulin expression and it therefore seems likely that β-tubulin depletion primarily at the cell cortex is the main driver behind the extra midline cell mitosis. Tubulin levels have been shown to influence RNA translation (Cleveland, 1989; Gonzalez-Garay and Cabral, 1995, 1996; Groisman et al., 2000), thus it is possible that changes in β-tubulin levels may stimulate release of G2 arrest posttranscriptionally. Disruption of cortical β-tubulin may also release a transcription factor tethered to the cell cortex, which after entering the nucleus activates the transcriptional cascade releasing cells into M-phase. Future experiments will test these two mechanisms. Our major finding is that disruption of microtubules, a basic component of every cell, can drive cells into mitosis. In animal models of tauopathies, ectopic expression of tau causes a breakdown of axonal microtubules (Cowan et al., 2010; Thies and Mandelkow, 2007) and it will be interesting to investigate if this disruption underlies the cell cycle activation of differentiated neurons in patients with tauopathy (Busser et al., 1998). The analogy between midline and vertebrate floor plate (Arendt and Nübler-Jung, 1999), and the conservation of the identified transcripts, *Jra* and *Miro*, lead us to suggest that the ventral midline may be used as an informative model to dissect the mechanisms directing mitotic activation during CNS damage or tissue homeostasis.

## Experimental Procedures

### Single Cell Transcriptome Amplification

We compared the transcriptome of the first sibling to the transcriptome of a single DiI labeled ventral ectodermal cell removed at the same time from the same embryo. The second, undamaged sibling was removed 30 min later and its transcriptome was compared to the same ectodermal transcriptome. The interval is shorter than the shortest time observed for entry into mitosis after damage (32 min), hence our analysis should exclude transcripts generally required for mitosis. This time interval also reduces the number of age specific differences between the cells. All three cells were collected from the same embryo to prevent differences due to genetic background or environmental factors. The experiments were repeated with a second embryo.

To calibrate the method, we pooled the transcriptome of four DiI labeled midline cells (stage 10) for reverse transcription and after digestion with RNaseH the volume was distributed into four separate aliquots. The aliquots were amplified with two different PCR enzymes and hybridized on microarrays. We obtained the highest correlation (92%) for the Long Expand Polymerase mix (Roche). We repeated this experiment using Long Expand Polymerase and amplifying through 20, 25, and 30 cycles. The results of the microarray hybridization indicate that 25 cycles are optimal. Only samples with a visible banding pattern on a 1% agarose gel yield a reproducible array pattern. Our initial tests created this protocol.

We freshly prepared 3 μl aliquots of the annealing mix, which consists of 0.3 μl of 10 pM anchored poly T primer (AAGCAGTGGTATCAACGCAGAGTACT_(26)_VN), 0.3 μl of 10 pM SM primer (AAGCAGTGGTATCAACGCAGAGTACGCrGrGrG), 0.4 μl of RNase inhibitor (Superase, Ambion), and 2 μl of Lysis Mix (4 μl of 10% NP40, 200 μl 0.1 M DTT and 796 μl of DEPC water). The 3 μl aliquots were kept on ice. Under fluorescent control (FITC filter) one single DiI labeled cell was removed from the embryo with a microcapillary. Special care was taken to only suck up fluorescent membrane and cytoplasm. 0.6 μl of the annealing mix was put on top of the fluorocarbon oil covering the embryos. The cell in the capillary was immediately expelled into the annealing mix and the drop on the fluorocarbon oil was removed under a dissecting scope and joined with the tube it had been taken from. Time between cell removal and release into the annealing mix was less than 1min. Samples were spun for 1 min at highest speed in a benchtop centrifuge and primers annealed for 3 min at 70°C in a PCR machine. Immediately after annealing the samples were frozen in a bath of dry ice/isopropanol. One microliter first strand buffer (Invitrogen), 0.5 μl 10 mM dNTP (NEB), and 0.5 μl reverse transcriptase (Superscript II, Invitrogen) were added to the frozen samples. Samples were thawed during spinning at maximum speed in a centrifuge, the samples were reverse transcribed at 37°C for 90 min in a PCR machine, followed by a heat inactivation of the enzyme at 65°C for 10 min. The RNA was digested with 0.25 μl RNaseH (Roche) for 20 min at 65°C. For PCR amplification, 2 μl primer (AAGCAGTGGTATCAACGCAGAGT), 5 μl buffer (Roche Applied), 2 μl 10 mM dNTP (NEB), 0.5 μl Polymerase Mix (Long Expand, Roche Applied), and 34.5 μl water were added. The PCR was set to one initial cycle of 95°C for 3 min, 5 min at 50°C, and 15 min at 68°C, followed by 24 cycles of 20 s at 95°C, 1 min at 60°C, and 7 min at 68°C.

## Supplemental Information

Document S1. Figures S1–S4 and Supplemental Experimental Procedures

Movie S1. Midline Divisions in Early Embryos 1, Related to Figure 1 Most midline cells divide once during early embryogenesis. Two midline precursors are labeled with the membrane dye DiI (red). The Jupiter::GFP fusion protein (green) labels microtubules (Morin et al., 2001). One frame documenting one Z level was recorded every minute. The movie consists of 240 frames covering 4 hr of development (stage 9 to late stage 11). Ventral view; anterior up. After approximately 4 hr, the upper midline cells divide again, whereas the cells at the bottom do not divide a second time. The upper cells differentiate into four VUM neurons, and the bottom cells develop into two midline glia (data not shown). We recorded the division of midline precursors in six embryos and never detected additional divisions before late stage 11.

Movie S2. Midline Divisions in Early Embryos 2, Related to Figure 1 The majority of midline precursors divide only once during early embryogenesis. Two midline precursors are labeled with the membrane dye DiI (red). The Jupiter::GFP fusion protein (green) labels microtubules (Morin et al., 2001). One frame documenting one Z level was recorded every 3 min. The movie consists of 107 frames covering 5 hr and 18 min of development (stage 9 to late stage 11). Ventral view; anterior up. The recording started at the end of the initial midline division. The upper midline cells disappear from focus. The cells at the bottom do not divide again. Both sibling pairs developed into two cell VUM clones (see Movie S3).

Movie S3. Differentiated Clones Derived from Precursors Documented in Movie S2, Related to Figure 1 Z sections of the two VUM clones derived from the midline cells documented in Movie S2. Ventral views; anterior up. Z sections start at the dorsal side of the nerve cord. Both clones comprise one VUM interneuron and one VUM motor neuron.

## References

[bib1] Arendt D., Nübler-Jung K. (1999). Comparison of early nerve cord development in insects and vertebrates. Development.

[bib2] Bedelbaeva K., Snyder A., Gourevitch D., Clark L., Zhang X.M., Leferovich J., Cheverud J.M., Lieberman P., Heber-Katz E. (2010). Lack of p21 expression links cell cycle control and appendage regeneration in mice. Proc. Natl. Acad. Sci. USA.

[bib3] Bergmann A., Tugentman M., Shilo B.Z., Steller H. (2002). Regulation of cell number by MAPK-dependent control of apoptosis: a mechanism for trophic survival signaling. Dev. Cell.

[bib4] Bossing T., Technau G.M. (1994). The fate of the CNS midline progenitors in *Drosophila* as revealed by a new method for single cell labelling. Development.

[bib5] Bossing T., Brand A.H. (2006). Determination of cell fate along the anteroposterior axis of the Drosophila ventral midline. Development.

[bib6] Busser J., Geldmacher D.S., Herrup K. (1998). Ectopic cell cycle proteins predict the sites of neuronal cell death in Alzheimer’s disease brain. J. Neurosci..

[bib7] Choksi S.P., Southall T.D., Bossing T., Edoff K., de Wit E., Fischer B.E., van Steensel B., Micklem G., Brand A.H. (2006). Prospero acts as a binary switch between self-renewal and differentiation in *Drosophila* neural stem cells. Dev. Cell.

[bib8] Cleveland D.W. (1989). Autoregulated control of tubulin synthesis in animal cells. Curr. Opin. Cell Biol..

[bib9] Cowan C.M., Bossing T., Page A., Shepherd D., Mudher A. (2010). Soluble hyper-phosphorylated tau causes microtubule breakdown and functionally compromises normal tau in vivo. Acta Neuropathol..

[bib10] Dong R., Jacobs J.R. (1997). Origin and differentiation of supernumerary midline glia in *Drosophila* embryos deficient for apoptosis. Dev. Biol..

[bib11] Dübel S., Schaller H.C. (1990). Terminal differentiation of ectodermal epithelial stem cells of Hydra can occur in G2 without requiring mitosis or S phase. J. Cell Biol..

[bib12] Egger B., Chell J.M., Brand A.H. (2008). Insights into neural stem cell biology from flies. Philos. Trans. R. Soc. Lond. B Biol. Sci..

[bib13] Galvin K.E., Ye H., Erstad D.J., Feddersen R., Wetmore C. (2008). Gli1 induces G2/M arrest and apoptosis in hippocampal but not tumor-derived neural stem cells. Stem Cells.

[bib14] Gonzalez-Garay M.L., Cabral F. (1995). Overexpression of an epitope-tagged beta-tubulin in Chinese hamster ovary cells causes an increase in endogenous alpha-tubulin synthesis. Cell Motil. Cytoskeleton.

[bib15] Gonzalez-Garay M.L., Cabral F. (1996). alpha-Tubulin limits its own synthesis: evidence for a mechanism involving translational repression. J. Cell Biol..

[bib16] Groisman I., Huang Y.-S., Mendez R., Cao Q., Theurkauf W., Richter J.D. (2000). CPEB, maskin, and cyclin B1 mRNA at the mitotic apparatus: implications for local translational control of cell division. Cell.

[bib17] Guo X., Macleod G.T., Wellington A., Hu F., Panchumarthi S., Schoenfield M., Marin L., Charlton M.P., Atwood H.L., Zinsmaier K.E. (2005). The GTPase dMiro is required for axonal transport of mitochondria to Drosophila synapses. Neuron.

[bib18] Holstein T.W., Hobmayer E., David C.N. (1991). Pattern of epithelial cell cycling in hydra. Dev. Biol..

[bib19] Jacobs J.R. (2000). The midline glia of *Drosophila:* a molecular genetic model for the developmental functions of glia. Prog. Neurobiol..

[bib20] Kernie S.G., Parent J.M. (2010). Forebrain neurogenesis after focal Ischemic and traumatic brain injury. Neurobiol. Dis..

[bib21] Khurana V., Feany M.B. (2007). Connecting cell-cycle activation to neurodegeneration in Drosophila. Biochim. Biophys. Acta.

[bib22] Klebes A., Knust E. (2000). A conserved motif in Crumbs is required for E-cadherin localisation and zonula adherens formation in Drosophila. Curr. Biol..

[bib23] Lehner C.F. (1991). Pulling the string: cell cycle regulation during *Drosophila* development. Semin. Cell Biol..

[bib24] Liu Y.P., Lang B.T., Baskaya M.K., Dempsey R.J., Vemuganti R. (2009). The potential of neural stem cells to repair stroke-induced brain damage. Acta Neuropathol..

[bib25] Michalopoulos G.K., DeFrances M.C. (1997). Liver regeneration. Science.

[bib26] Raivich G., Behrens A. (2006). Role of the AP-1 transcription factor c-Jun in developing, adult and injured brain. Prog. Neurobiol..

[bib27] Raivich G., Bohatschek M., Da Costa C., Iwata O., Galiano M., Hristova M., Nateri A.S., Makwana M., Riera-Sans L., Wolfer D.P. (2004). The AP-1 transcription factor c-Jun is required for efficient axonal regeneration. Neuron.

[bib28] Scharfman H.E., Gray W.P. (2007). Relevance of seizure-induced neurogenesis in animal models of epilepsy to the etiology of temporal lobe epilepsy. Epilepsia.

[bib29] Schmidt T., David C.N. (1986). Gland cells in Hydra: cell cycle kinetics and development. J. Cell Sci..

[bib30] Stemerdink C., Jacobs J.R. (1997). Argos and Spitz group genes function to regulate midline glial cell number in *Drosophila* embryos. Development.

[bib31] Tanaka E.M., Gann A.A., Gates P.B., Brockes J.P. (1997). Newt myotubes reenter the cell cycle by phosphorylation of the retinoblastoma protein. J. Cell Biol..

[bib32] Thies E., Mandelkow E.M. (2007). Missorting of tau in neurons causes degeneration of synapses that can be rescued by the kinase MARK2/Par-1. J. Neurosci..

[bib33] Wheeler S.R., Stagg S.B., Crews S.T. (2008). Multiple Notch signaling events control Drosophila CNS midline neurogenesis, gliogenesis and neuronal identity. Development.

