# Supplementary material for: Disruption of Microtubule Integrity Initiates Mitosis during CNS Repair
Source: Dev Cell. 2012 Aug 14;23(2):433–40. doi: 10.1016/j.devcel.2012.06.002 (PMC3420022; doi:10.1016/j.devcel.2012.06.002)
Supplement: Document S1. Figures S1–S4 and Supplemental Experimental Procedures [file mmc1.pdf]

Developmental Cell, Volume *23*

Supplemental Information

Disruption of Microtubule Integrity Initiates Mitosis during CNS Repair

Torsten Bossing, Claudia S. Barros, Bettina Fischer, Steven Russell, and David Shepherd

Supplemental Information Inventory

Figure S1. Differentiated midline siblings lose their membrane connection and repair capacity. Related to Figure 1.

Figure S2. Loss of the ectodermal attachment does not trigger midline cell divisions. Related to Figure 2.

Figure S3. Actin depolymerisation or microtubule stabilisation by Taxol fail to force midline cells into M-phase. Related to Figure 2.

Figure S4. Ectopic Miro expression in midline cells can prevent damage repair and loss of Jra does not increase midline cell division without damage. Related to Figure 3 and 4.

Supplementary Experimental Procedures

Supplementary References

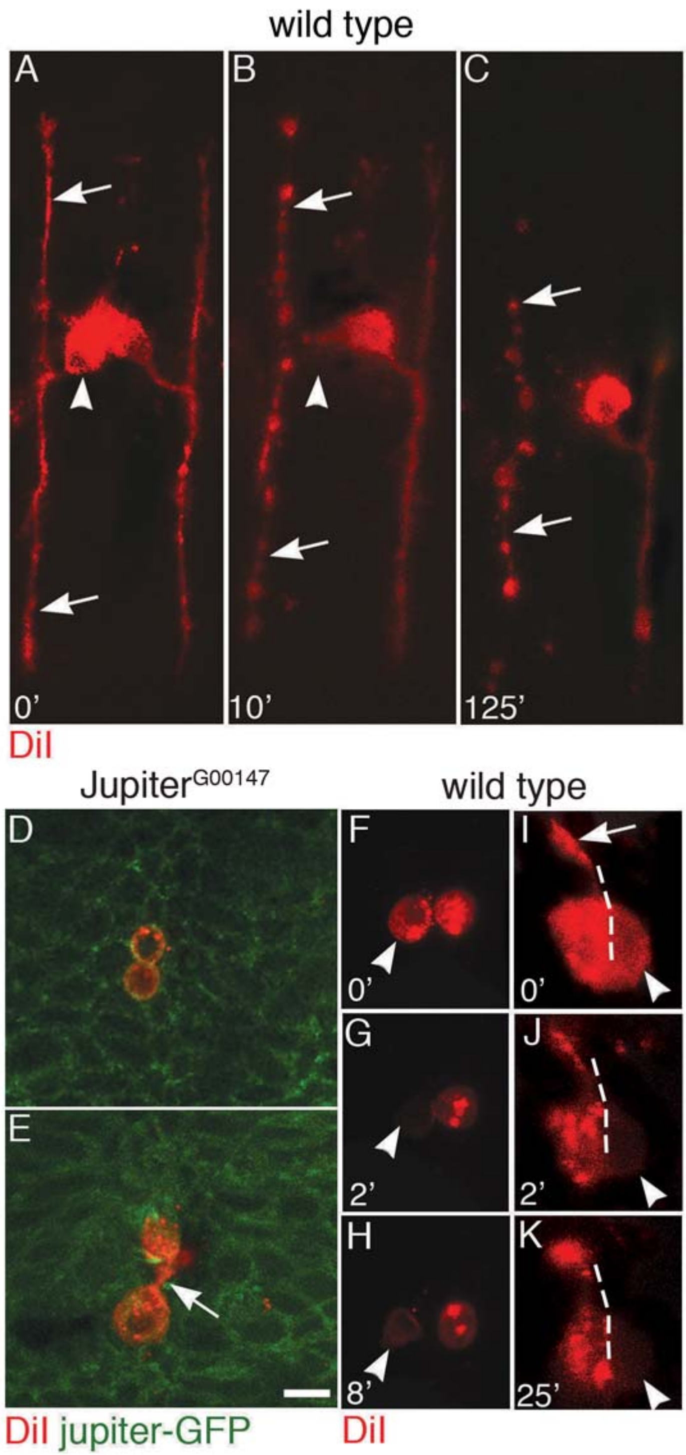

Figure S1, Bossing et al.

Figure S1. Differentiated midline siblings lose their membrane connection and repair capacity. Related to Figure 1. Genotypes indicated at top of panels. Ventral views, anterior up. scale, 5 µm. (A - C) One sibling (arrowhead) is removed after axon extension (arrows). The surviving sibling is unable to replace the ablated cell. Time given in minutes after ablation. (D, E) Two midline siblings (D) were mechanically separated to reveal a connection stained by the membrane dye DiI (red, E, arrow). Cell outlines labelled by GFP (green) gene trap *(*$Jupiter^{G00174}$*)*. (F - H) After bleaching, an undifferentiated sibling (arrowhead) always regains fluorescence (n=8). (I - K) After axon extension (arrow), a bleached midline sibling (arrowhead) never recovers fluorescence indicating a loss of the membrane connection (n=7). Cells are labelled with membrane dye DiI. Time given in minutes after bleaching. The connection between both cells seems not to originate from an incomplete cytokinesis because we cannot discover actin rings (Figure S3A, B, red), Anillin (Field et al., 2005) or Pavarotti (Adams et al., 1998) accumulation between sibling cells (data not shown).

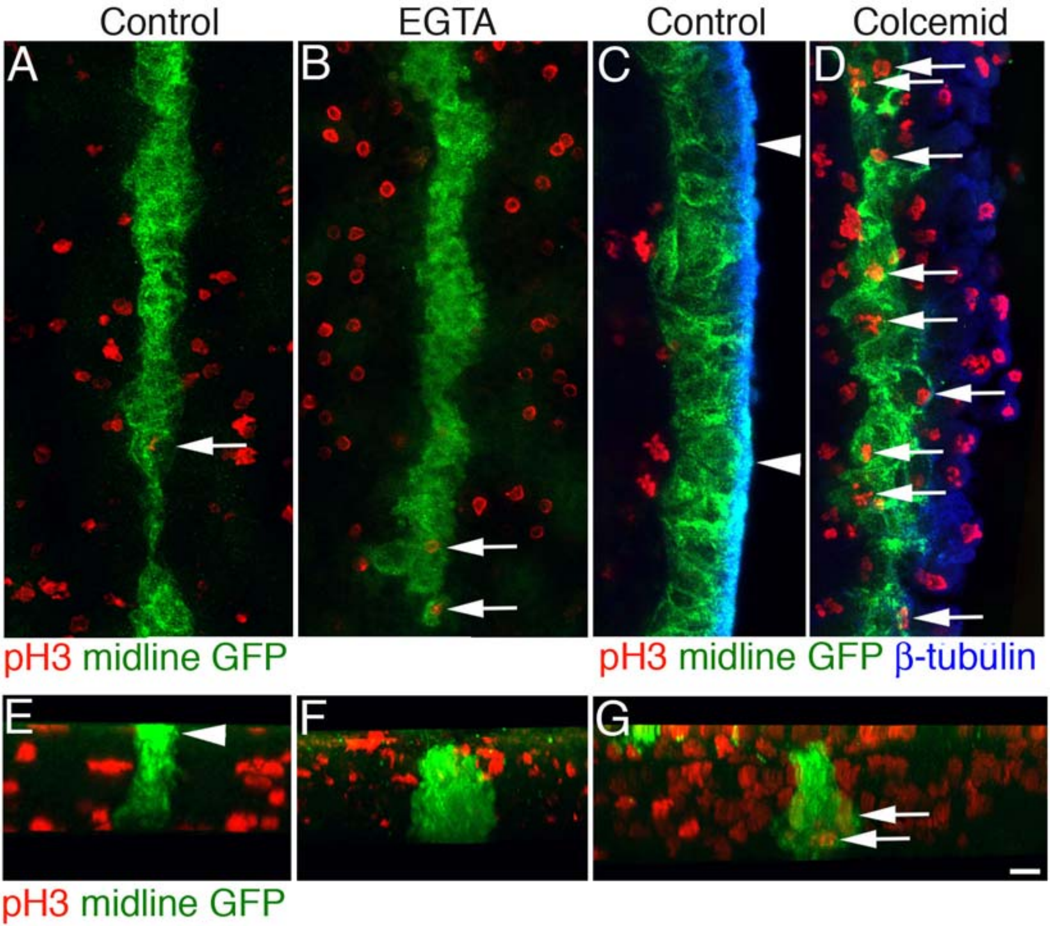

Figure S2, Bossing et al.

Figure S2. Loss of the ectodermal attachment does not trigger midline cell divisions. Related to Figure 2. Genotype of all embryos is *sim::GAL4/ UAS::CD8-GFP; sim::GAL4/* +. Ventral views (A, B), lateral views (C, D) or transverse sections (E, F, G) of stage 10 embryos are shown. Anterior up. Scale, 5µm. (A, E) In water injected embryos, delaminating midline cells (green) extend apical processes to the ectoderm (arrowhead) and the cells rarely divide (arrow, red, pH3). (B, F) Injection of EGTA interferes with Cadherin adhesion by depleting Calcium ions. The loss of Cadherin adhesion does not induce extra cell divisions at the midline (arrows, red, B) but midline cells fall into the embryo (F). (C) In water injected stage 10 embryos, midline cells (green) show an accumulation of microtubules (blue) in the apical extensions (arrowheads) and no divisions (red, pH3) can be detected. (D, G) Injection of colcemid depolymerises microtubules (blue, β-tubulin) causing the collapse of the apical extensions and the fall of the midline cells into the embryo (G). In addition, midline cells enter into mitosis (arrows, red, D, G).

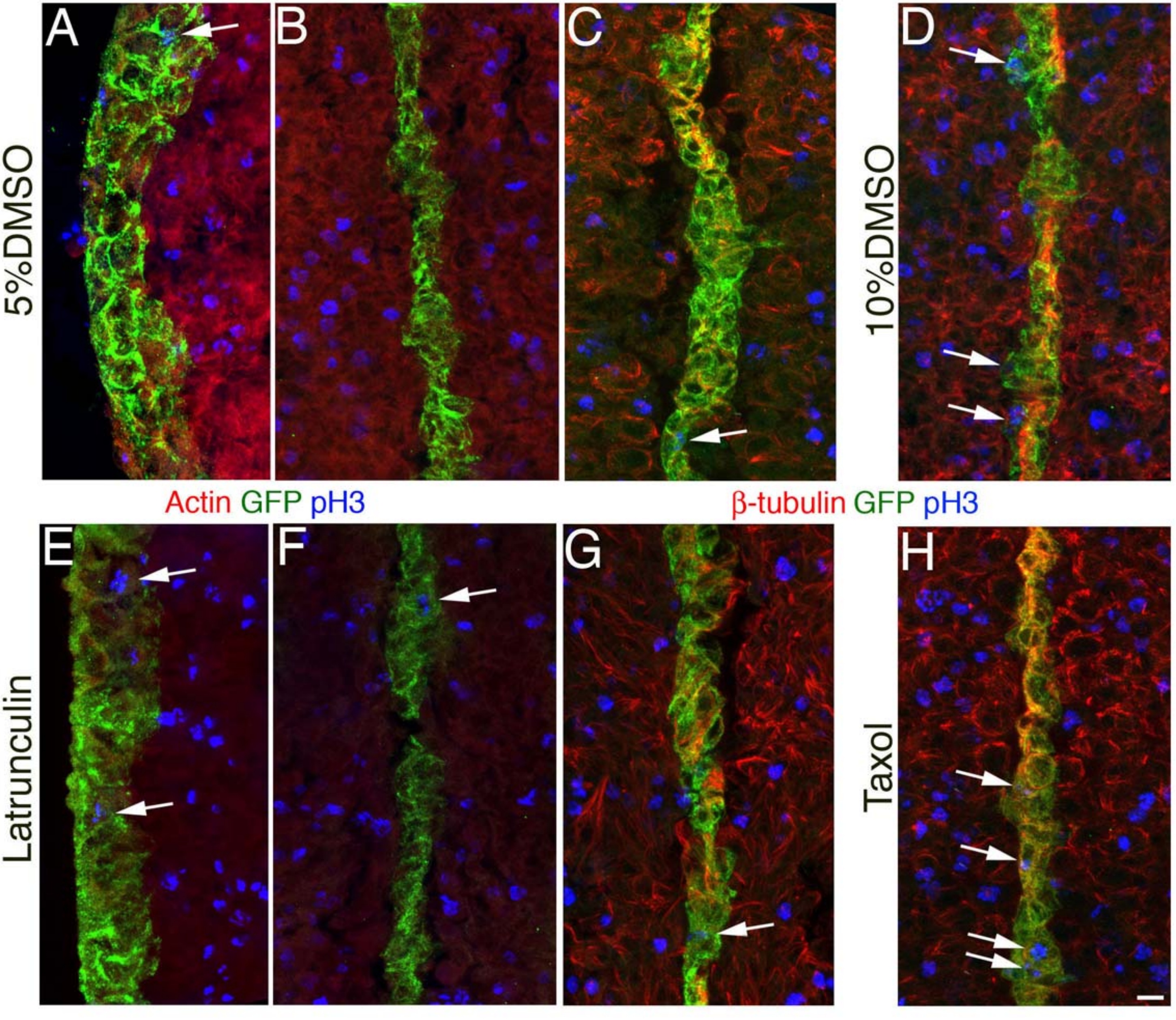

Figure S3, Bossing et al.

Figure S3. Actin depolymerisation or microtubule stabilisation by Taxol fail to force midline cells into M-phase. Related to Figure 2. Genotype of all embryos is *sim::GAL4/ UAS::CD8-GFP; sim::GAL4/ +*. Lateral views (A, E) and ventral views (B-D, F-H) of stage 11 are shown. Anterior up. Scale, 5µm. (A, B, C, D) Control injected embryos. Injection of 5% DMSO (A-C) or 10% DMSO (D) does not interfere with Actin polymerisation (red, A, B) but slightly disrupts the microtubule cytoskeleton (red, compare C and D). In DMSO injected embryos up to six dividing cells (arrows, blue, pH3) in the ventral midline (green) can be found (C, D). Disruption of the actin cytoskeleton (red) by Latrunculin A injection (n=12) neither causes midline cells to fall into the embryos (E) nor does it increase midline cell divisions (arrow, blue, F). (G) Injection of Latrunculin A does not depolymerise microtubule fibres (red). (H) Stabilisation of microtubules by taxol injections (n =14) results in the metaphase arrest of the regular cell divisions outside the midline. Compared to control injected embryos (D), there is no increase in mitotic midline cells.

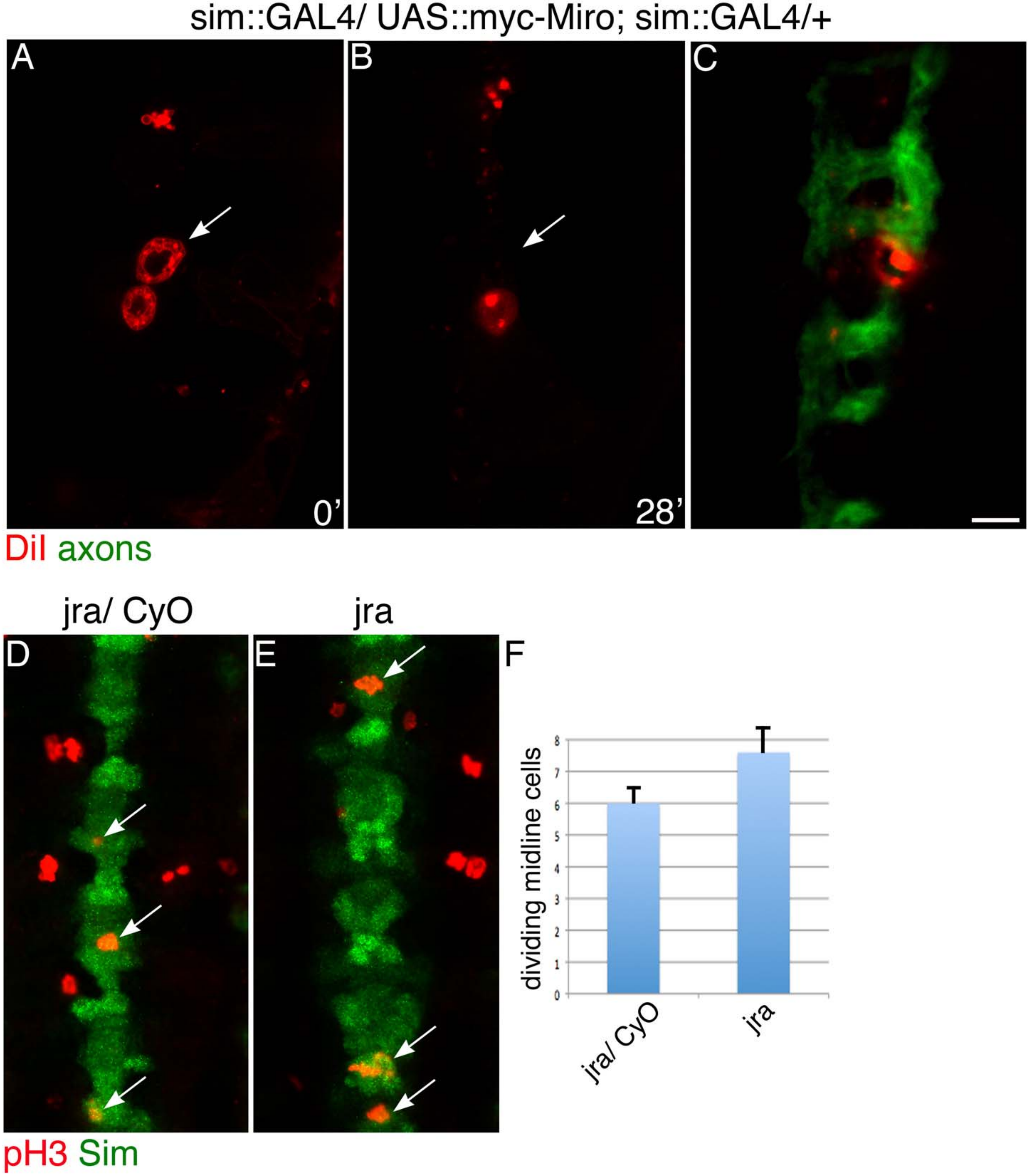

Figure S4, Bossing et al.

Figure S4. Ectopic Miro expression in midline cells can prevent damage repair and loss of Jra does not increase midline cell division without damage. Related to Figure 3 and 4. Genotypes indicated at top of panel. Ventral view; Anterior up. Scale, 6μm (A, B) A midline sibling (red) in an embryo expressing Miro in all midline cells was ablated (arrow). Time in minutes after ablation. (C) In stage 17, the surviving sibling (DiI, red) did not divide again. Axons are labelled green (BP102). (D, E) In stage 13 embryos, a similar number of midline cells (green, Sim) enter into division (arrows, red, pH3) in heterozygous (D) and homozygous *jra* mutants (E). (F) Number of dividing midline cells in heterozygous (jra/ CyO, n = 10) and homozygous (jra, n = 8) mutants. ttest; no significance. Error Bars, S.E.M

Supplemental Experimental Procedures

Single cell transcriptome microarray labelling and hybridisation

The control ventral ectoderm cell sample was labelled as 4 technical replicates with Cy3 and the duplicate midline sibling cells with Cy5: 1μg of amplified DNA was labelled with the BioPrime DNA labelling kit (Invitrogen) in the presence of fluorescently labelled Cy3- or Cy5-cCTP (GE Healthcare) at 37°C for 2 hours. The labelled samples were purified with Sephadex G50 columns (GE Healthcare). Microarrays containing long oligonucleotides (GEO platform accession GPL5135) were printed in house using a QArray2 (Genetix) spotter on FMB PowerMatrix slides. Co-hybridisation of labelled ventral ectoderm cell derived DNA along with DNA derived from the surviving sibling cell was performed for 16 hours at 51°C in a GeneTac hybridisation station (Digilab Genomic Solutions Inc) on 4 arrays. Post hybridisation washes were performed according to the slide manufacturer's recommendation. Detailed protocols for array spotting, labelling, hybridisation and washing are available at http://www.flychip.org.uk/protocols/.

Microarray analysis

Arrays were scanned at 5μm resolution using individually optimised PMT gain settings for each channel in a GenePix 4000B dual laser scanner (Axon Instruments). Raw intensity values were extracted from the images using Dapple (Buhler et al., 2000). One array was excluded from further analysis based on the results of the QC-plots of the raw data. The raw intensity values of the remaining 3 arrays were filtered to retain only genes that were accepted on at least one of the arrays and with

raw intensity values above 50 for all arrays (2406 genes remained). The data was normalised using a quantile method (Bolstad et al., 2003).

Fly strains

The wild type strain *Oregon R* was used for most of the ablations and also for colcemid injections. For all other drug injections we used *sim-Gal4/ UAS::CD8-GFP; sim-Gal4/+*. The strain jupiter^{G00147}, a GFP gene trap into *jupiter* (Morin et al., 2001), outlines all embryonic cells and was used to follow adjacent cells after midline cell ablation. Embryos maternally and zygotically mutant for *myospheroid (mys*^{XG43}*, βPS-Integrin)* were a kind gift from Maria D. Martin-Bermudo. In addition, we used the transformant flies *w1118;p[w*$^{+}$ *Ub-GFP-Pav-KLP]53* which ubiquitously express a GFP-Pavarotti fusion protein (Minestrini et al., 2002), the E-Cadherin mutants *FRT913 shg*^{IH} *bw sp/ Cyo p[ftz-lacZ]* (Tepass et al., 1996) and the Jra mutant *Jra*^{IA109}.

GAL4 transformants and UAS transformants used are *w; sim-GAL4/ Cyo; sim-GAL4/ sim-GAL4* (Scholz et al., 1997), *rhomboid-GAL4* (Klaes et al., 1994), *w; UAS-Crumbs* (Wodarz et al., 1993), *y w; UAS- human histone H2B-YFP* (Bellaiche et al., 2001), *yw; GAL4*^{V2h} (Hacker et al., 1997), *UAS-Miro*$^{RNAi(TRIP\ JF02775)}$ (Bloomington); *UAS-Miro*$^{RNAi\ (106683)}$ (Vienna Drosophila RNAi Centre).

Immunohistochemistry and in situ hybridisation

Immunohistochemistry has been described previously (Bossing et al., 1996) with the exception of anti-β−tubulin stainings. The anti-β−tubulin antibody (E7, DSHB) was

diluted 1:10 in PBT (PBS with 1% Triton X100 added). For phalloidin stainings embryos were fixed like previously described (Bossing et al., 2002) and the vitelline membrane was removed manually. After removal of the vitelline membrane embryos were fixed with 4% FA in PBS for 10min. Embryos were incubated with 2u (66nM) Phalloidin-Alexa568 (Molecular Probes) diluted in 200µl PBT (PBS with 0.3% Triton added) for 2 h at room temperature.

The following antibodies were used: anti-α-tubulin (clone DM1A, Sigma); anti-BrdU, 1:1000 (clone BU33, Sigma); anti-β-tubulin (clone E7, DSHB); anti-cyclinB, 1:1000 (kindly provided by C. Lehner; Lehner and O`Farrell, 1990); anti-Crumbs, 1:5 (Tepass and Knust, 1993); anti-DE Cadherin, 1:50 (Uemura et al., 1996); anti-Digoxigenin, 1:2000 (coupled to alkaline phosphatase; Roche); anti-GFP, 1:2000 (rabbit polyclonal, abcam); anti-GFP, 1:10 (mouse monoclonal 3E6, Molecular Probes); anti-phosphorylated histone H3, 1:2000 (Upstate); anti-single minded, 1:3 (mouse, DSHB); anti-single minded, 1:200 (rat, kindly provided by S.Crews; Ward et al., 1998). Secondary antibodies used were coupled to biotin, alkaline phosphatase, Cy5 (Jackson Laboratories), Alexa488, Alexa568 (Molecular Probes).

The in situ hybridisation protocol has been described previously (Bossing and Brand, 2002). We first generated a *string*-T7 or *jra*-T7 cDNA by PCR and used the T7 coupled templates for in-vitro transcription (T7 polymerase, Roche Applied) of a riboprobe incorporating Digoxigenin-UTPs (DIG RNA Labelling Mix, Roche Applied). Immunohistochemistry was done after development of the in situ signal using a TSA kit (Amersham). All embryos were mounted as flat preparations in 70%

glycerol/ 30% Vectashield (Vector Labs). Images were collected on a Zeiss LSM710 and assembled in Photoshop CS3.

Embryo injections

All drugs were injected into the perivitelline space. The injected volume was equal to about 1% of the total embryonic volume. Injection of 3mg/ ml (8mM) Colcemid is sufficient to partially depolymerise the microtubule cytoskeleton in midline cells as revealed by anti-tubulin stainings. We also injected 100ug/ ml and 1mg/ ml of the microtubules depolymerising drug Vinblastine (Sigma). 100ug/ ml is sufficient to depolymerise the tubulin cytoskeleton. Antibodies against DE-Cadherin show that injection of 760mg/ ml (2M) EGTA in water destroys the apical Cadherin network. Water was injected as control for colcemide and EGTA injections. LatrunculinA (Sigma) was injected at the concentration of 0.1mg/ ml (25mM) in 5% DMSO/ water. Depolymerisation of the actin cyctoskeleton was examined with Phalloidin-Alexa568. Injections of 5% DMSO in water served as control. Microtubules were stabilised by the injection of 1.25mg/ ml (1.5mM) Taxol in 10% DMSO/ water. BrdU (Sigma), at a concentration of 15mg/ ml (50mM) in water, was injected directly into the syncytial blastoderm shortly before the onset of gastrulation (150-170 min after fertilisation, stage 5). All embryos were fixed and devitellinised as described previously (Bossing et al., 2002). Before the anti-BrdU incubation the embryos were hydrolyzed for 30min with 2N HCl at room temperature. The anti-BrdU staining was developed before incubation with the second primary antibody.

Generation of transgenic flies

The cDNAs of α-tubulin 67C and β-tubulin 56D without untranslated 5' or 3' ends were amplified from an embryonic cDNA pool adding suitable restriction sites. Both tubulins were cloned into a pUASp vector which has GFP incorporated 5' from the MCS. Both tubulins have been selected because of their CNS expression in wild type embryos. Constructs were injected into *yw; P(ry,* D*2-3), Sb/TM6, Ubx* embryos (Robertson et al., 1988).

Cell labelling, cell ablation and FRAP

Single cell labellings with DiI (Molecular Probes) were performed as described previously (Bossing and Technau, 1994; Bossing et al., 1996).

Cell labelling and ablation was carried out with an inverted microscope using a 100x oil immersion objective. The ablation capillary has an outer diameter of about 3μm and a tip bevelled at an angle of 25°. The perivitelline membrane was pierced with a capillary along the edge formed by its adhesion to the glue-coated coverslip. The opening was positioned at the anterior-posterior position of the cell targeted for ablation. With slight movements of the microscope table the hole in the membrane was widened. Great care was taken to avoid any contact between tissue and capillary in these preparatory steps. The capillary was steered inside the perivitelline space, above the ectoderm, towards the targeted cell. When the tip reached the cell, the capillary was lowered into the tissue, pushing away the ectodermal cells. The ablation was done under fluorescence control [maximal 15 seconds of excitation (450-490nm) using a 50Watt Halogen lamp as a source].

For FRAP experiments we used a Zeiss LSM710. The bleaching was set to 6 Z-levels 1μm each and stopped after the fluorescence reached 5% of original intensity.

Supplemental References

Adams, R.R., Tavares, A.A., Salzberg, A., Bellen, H.J., and Glover, D.M. (1998). pavarotti encodes a kinesin-like protein required to organize the central spindle and contractile ring for cytokinesis. Genes Dev *12*, 1483-1494.

Bellaiche, Y., Gho, M., Kaltschmidt, J.A., Brand, A.H., and Schweisguth, F. (2001). Frizzled regulates localization of cell-fate determinants and mitotic spindle rotation during asymmetric cell division. Nat Cell Biol *3*, 50-57.

Bolstad, B.M., Irizarry, R.A., Astrand, M., and Speed, T.P. (2003). A comparison of normalization methods for high density oligonucleotide array data based on variance and bias. Bioinformatics *19*, 185-193.

Bossing, T., Barros, C.S., and Brand, A.H. (2002). Rapid tissue-specific expression assay in living embryos. Genesis *34*, 123-126.

Bossing, T., and Brand, A.H. (2002). Dephrin, a transmembrane ephrin with a unique structure, prevents interneuronal axons from exiting the Drosophila embryonic CNS. Development *129*, 4205-4218.

Bossing, T., and Technau, G.M. (1994). The fate of the CNS midline progenitors in *Drosophila* as revealed by a new method for single cell labelling. Development *120*, 1895-1906.

Bossing, T., Udolph, G., Doe, C.Q., and Technau, G.M. (1996). The embryonic central nervous system lineages of *Drosophila melanogaster* I. Neuroblast lineages derived from the ventral half of the neuroectoderm. Dev Bio *179*, 41-64.

Buhler, J., Ideker, T., and Haynor, D. (2000). Dapple: Improved techniques for finding spots on DNA microarrays. UW CSE Technical Report *UWTR 2000-08-05*.

Field, C.M., Coughlin, M., Doberstein, S., Marty, T., and Sullivan, W. (2005). Characterization of anillin mutants reveals essential roles in septin localization and plasma membrane integrity. Development *132*, 2849-2860.

Hacker, U., Lin, X., and Perrimon, N. (1997). The *Drosophila sugarless* gene modulates Wingless signaling and encodes an enzyme involved in polysaccharide biosynthesis. Development *124*, 3565-3523.

Klaes, A., Menne, T., Stollewerk, A., Scholz, H., and Klambt, C. (1994). The Ets transcription factors encoded by the *Drosophila* gene *pointed* direct glial cell differentiation in the embryonic CNS. Cell *78*, 149-160.

Lehner, C.F., and O`Farrell, P.H. (1990). The roles of *Drosophila* Cyclins A and B in mitotic control. Cell *61*, 535-547.

Minestrini, G., Mathe, E., and Glover, D.M. (2002). Domains of the Pavarotti kinesin-like protein that direct its subcellular distribution: effects of mislocalisation on the tubulin and actin cytoskeleton during Drosophila oogenesis. J Cell Sci *115*, 725-736.

Morin, X., Daneman, R., Zavortink, M., and Chia, W. (2001). A protein trap strategy to detect GFP-tagged proteins expressed from their endogenous loci in Drosophila. Proc Natl Acad Sci U S A *98*, 15050-15055.

Robertson, H.M., Preston, C.R., Phillis, R.W., Johnson-Schlitz, D., Benze, W.R., and Engels, W.R. (1988). A stable source of P-element transposase in *Drosophila melanogaster*. Genetics *118*, 461-470.

Scholz, H., Sadlowski, E., Klaes, A., and Klambt, C. (1997). Control of midline glia development in the embryonic *Drosophila* CNS. Mech Dev *62*, 79-91.

Tepass, U., Gruszynski-DeFeo, E., Haag, T.A., Omatyar, L., Torok, T., and Hartenstein, V. (1996). shotgun encodes Drosophila E-cadherin and is preferentially required during cell rearrangement in the neurectoderm and other morphogenetically active epithelia. Genes Dev *10*, 672-685.

Tepass, U., and Knust, E. (1993). Crumbs and stardust act in a genetic pathway that controls the organization of epithelia in Drosophila melanogaster. Dev Biol *159*, 311-326.

Uemura, T., Oda, H., Kraut, R., Hayashi, S., Kotaoka, Y., and Takeichi, M. (1996). Zygotic Drosophila E-cadherin expression is required for processes of dynamic epithelial cell rearrangement in the Drosophila embryo. Genes Dev *10*, 659-671.

Ward, M.P., Mosher, J.T., and Crews, S.T. (1998). Regulation of bHLH-PAS protein subcellular localization during Drosophila embryogenesis. Development *125*, 1599-1608.

Wodarz, A., Grawe, F., and Knust, E. (1993). *Crumbs* is involved in the control of apical protein targeting during *Drosophila* epithelial development. Mech Dev *44*, 175-187.
