# Supplementary figures and images for: Disruption of Microtubule Integrity Initiates Mitosis during CNS Repair
Source: Dev Cell. 2012 Aug 14;23(2):433–40. doi: 10.1016/j.devcel.2012.06.002 (PMC3420022; doi:10.1016/j.devcel.2012.06.002)

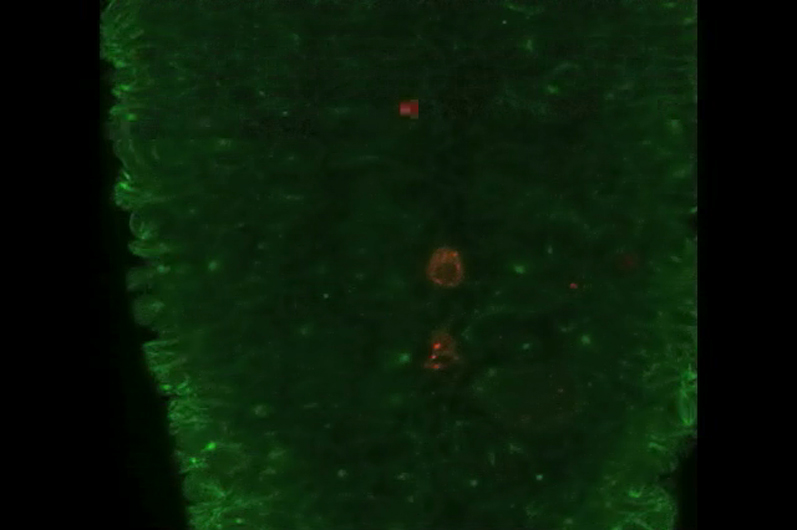

Supplement: Movie S1. Midline Divisions in Early Embryos 1, Related to Figure 1 — Most midline cells divide once during early embryogenesis. Two midline precursors are labeled with the membrane dye DiI (red). The Jupiter::GFP fusion protein (green) labels microtubules (Morin et al., 2001). One frame documenting one Z level was recorded every minute. The movie consists of 240 frames covering 4 hr of development (stage 9 to late stage 11). Ventral view; anterior up. After approximately 4 hr, the upper midline cells divide again, whereas the cells at the bottom do not divide a second time. The upper cells differentiate into four VUM neurons, and the bottom cells develop into two midline glia (data not shown). We recorded the division of midline precursors in six embryos and never detected additional divisions before late stage 11. [file mmc2.jpg]

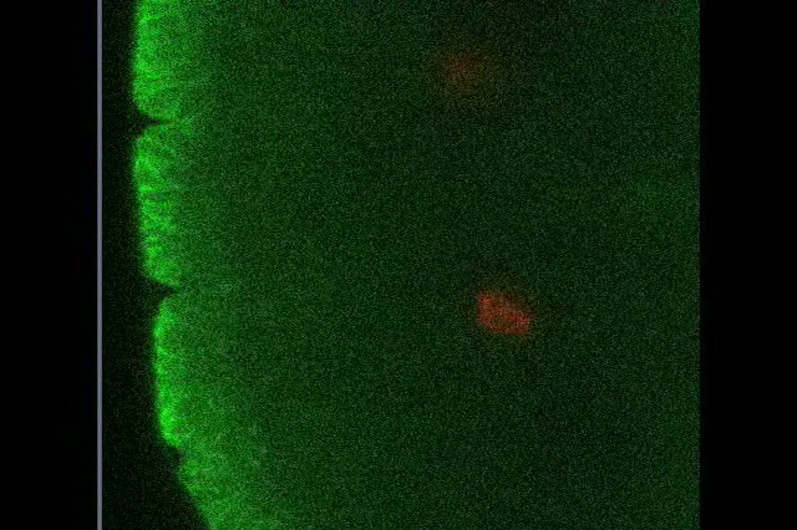

Supplement: Movie S2. Midline Divisions in Early Embryos 2, Related to Figure 1 — The majority of midline precursors divide only once during early embryogenesis. Two midline precursors are labeled with the membrane dye DiI (red). The Jupiter::GFP fusion protein (green) labels microtubules (Morin et al., 2001). One frame documenting one Z level was recorded every 3 min. The movie consists of 107 frames covering 5 hr and 18 min of development (stage 9 to late stage 11). Ventral view; anterior up. The recording started at the end of the initial midline division. The upper midline cells disappear from focus. The cells at the bottom do not divide again. Both sibling pairs developed into two cell VUM clones (see Movie S3). [file mmc3.jpg]

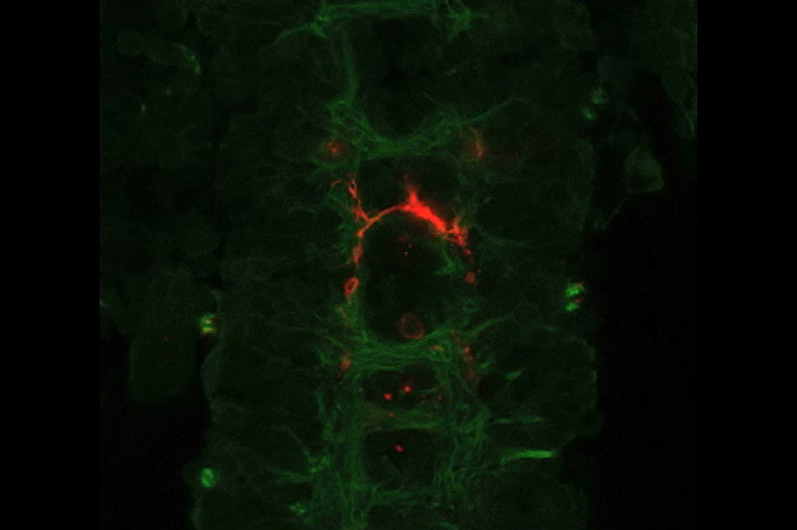

Supplement: Movie S3. Differentiated Clones Derived from Precursors Documented in Movie S2, Related to Figure 1 — Z sections of the two VUM clones derived from the midline cells documented in Movie S2. Ventral views; anterior up. Z sections start at the dorsal side of the nerve cord. Both clones comprise one VUM interneuron and one VUM motor neuron. [file mmc4.jpg]
